# Micro-Area Ferroelectric, Piezoelectric and Conductive Properties of Single BiFeO_3_ Nanowire by Scanning Probe Microscopy

**DOI:** 10.3390/nano9020190

**Published:** 2019-02-02

**Authors:** Shenglan Wu, Jing Zhang, Xiaoyan Liu, Siyi Lv, Rongli Gao, Wei Cai, Fengqi Wang, Chunlin Fu

**Affiliations:** 1 School of Metallurgy and Materials Engineering, Chongqing University of Science and Technology, Chongqing 401331, China; shenglanwu@126.com (S.W.); m17725020266_1@163.com (J.Z.); lsy3134486991@163.com (S.L.); gaorongli2008@163.com (R.G.); caiwei_cqu@163.com (W.C.); wangle_17@126.com (F.W.); 2 Chongqing Key Laboratory of Nano/Micro Composite Materials and Devices, Chongqing 401331, China

**Keywords:** bismuth ferrite, nanowire, micro-area, SPM

## Abstract

Ferroelectric nanowires have attracted great attention due to their excellent physical properties. We report the domain structure, ferroelectric, piezoelectric, and conductive properties of bismuth ferrite (BFO, short for BiFeO_3_) nanowires characterized by scanning probe microscopy (SPM). The X-ray diffraction (XRD) pattern presents single phase BFO without other obvious impurities. The piezoresponse force microscopy (PFM) results indicate that the nanowires possess a multidomain configuration, and the maximum piezoelectric coefficient (*d*_33_) of single BFO nanowire is 22.21 pm/V. Poling experiments and local switching spectroscopy piezoresponse force microscopy (SS-PFM) demonstrate that there is sufficient polarization switching behavior and obvious piezoelectric properties in BFO nanowires. The conducting atomic force microscopy (C-AFM) results show that the current is just hundreds of pA at 8 V. These lay the foundation for the application of BFO nanowires in nanodevices.

## 1. Introduction

Multiferroic materials, which exhibit both ferroelectric and ferromagnetic properties simultaneously as well as magnetoelectric coupling effects, have been intensively studied due to their potential applications in four-stage logic memory devices, magnetoelectric random access memory (MeRAM) [1], magnetic field sensors, energy harvester, magnetoelectric resonator, and read head [2,3,4,5,6]. Single-phase multiferroic BiFeO_3_ (BFO) is one of the most studied lead-free multiferroic materials, due to its high ferroelectric Curie (*T*_C_ = 1103 K), Neel (*T*_N_ = 643 K) temperatures, and large ferroelectric polarization of 100 μC/cm^2^ [7]. The main reason for ferroelectricity generation of BFO is that 6s^2^ of Bi^3+^ is hybridized with 6p vacant orbital or 2p of O^2−^ orbital, and the asymmetric center distortion caused by electron cloud. The polarization direction follows the body diagonal direction <111> [8]. The 71°, 109°, and 180° domain walls were found in BFO material in previous research [9].

Compared with bulk counterparts, one-dimensional structures (nanotubes [10,11], nanorods [12,13], nanowires [14,15], etc.) that have surface effects, size effects, and macroscopic quantum tunneling effects offer prospects for enhancing the electrical, thermal, and mechanical properties of a broad range of functional materials and composites [16,17,18,19,20], and play a momentous role in the next generation of electron devices. Particularly, one-dimensional semiconductor nanowires (NWs) have been envisioned as a candidate for efficient and small devices [21]. The BFO NWs have recently been studied extensively in the quest to miniaturize devices, and these studies found that the BFO NWs have highly efficient carrier separation [19], negative thermal quenching of emission [22], extraordinary electrostatic response [23], and remarkably high polarization [24]. However, most of the research focuses on macroscopic properties and studies on the domain structure of single NW are still lacking. Recently, interesting physical phenomena at the nanoscale have been discovered in BFO NWs, such as magnetoelectric (ME) coupling [25], surface dominant sub-bandgap photocarrier generation [26], and different PFM signals from one NW to another [27]. Domain structure is an important part of BFO materials, and plays a significant role in determining the performance. Hence, it is necessary to research the domain structure and micro-zone properties of NWs.

In this paper, BFO NWs were prepared by hydrothermal method, and the micro-zone performances, such as ferroelectric, piezoelectric, and conductive properties of single BFO NW were investigated by scanning probe microscopy (SPM), which lays a solid foundation for the application of BFO NWs in nanodevices.

## 2. Materials and Methods

All chemicals were used as received, and the purity of the products exceeded 99%. BiFeO_3_ NWs were prepared by hydrothermal method. The details of the preparation method are as follows. Firstly, bismuth nitrate (Bi(NO_3_)_3_·9H_2_O) and ferric chloride (FeCl_3_·6H_2_O) in a 1:1 stoichiometric ratio were dissolved in 50 mL acetone to form a solution. Secondly, 200 mL deionized water was added into the solution, then, sodium hydroxide (NaOH) solution was slowly added to the above solution to allow precipitation of Fe^3+^ and Bi^3+^ ions by constant stirring, whereby a brown precipitate was formed. The brown sediment was washed several times with deionized water and centrifuged out, repeatedly, until pH = 7. Next, NaOH solution was added to the brown precipitate and stirred for 30 min at room temperature. Then, the solution was transferred to a reactor with tetrafluoroethylene lining, and heated at 200 °C for 78 h. The brown powder that was obtained after being washed with deionized water and ethanol was dried at 60 °C.

The BFO NW powder was dispersed evenly in ethylene glycol solvent by ultrasonic waves. A small amount of supernatant was dropped into the cleaned fluorine-doped tin oxide (FTO) glass substrate by pipette. Finally, tube furnace was used for annealing treatment at 300 °C for 6 h.

X-ray diffraction (XRD, SmartLab-9, Japan) was utilized to determine the crystal structure using Cu *Kα* radiation (*λ* = 1.5406 Å) in the 2*θ* range, from 20° to 80°. Detailed analysis of microstructure and micro-zone performances was performed using scanning probe microscopy (SPM, Cypher, America). The experiment was done at room temperature and 50% humidity. The AFM probe was silicon and was coated by Pt. The spring constant of the cantilevers was *k* = 0.03–0.2 N/m. The resonant frequency was 14–28 kHz. The amplitude of the AC voltage applied ranged from 4.0 V to 5.5 V.

## 3. Results and Discussion

### 3.1. Crystal Structure

**Figure 1:**
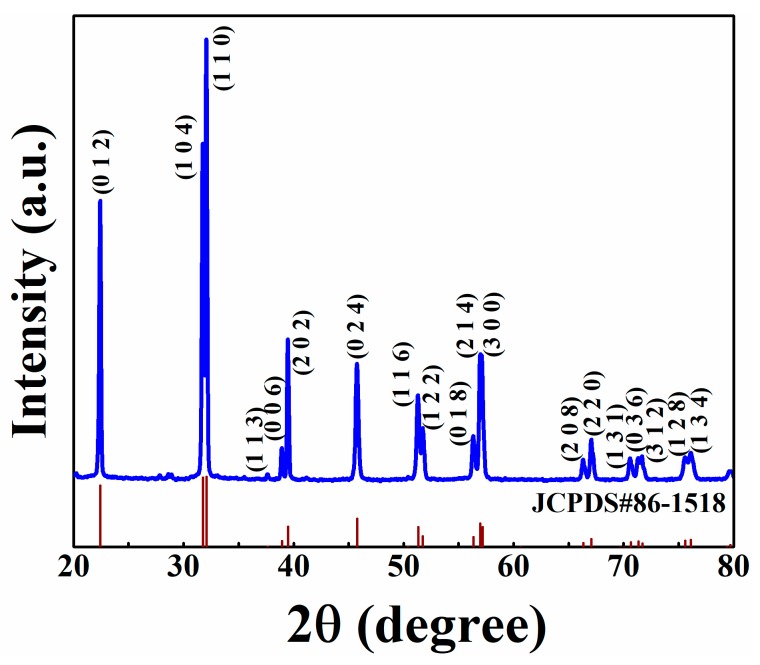
XRD pattern of BiFeO_3_ (BFO) powders.

Figure 1 shows XRD patterns of BFO powders prepared by the hydrothermal process after annealing treatment. It can be seen that the sample can be indexed in a single BFO phase without any impurities for the 2*θ* angle, from 20° to 80°, which belong to a hexagonal with *R*3*c* space group (JCPDS No. 86-1518). It shows a well-crystallized phase that represents three diffraction peaks at 22.6°, 31.8°, and 32.1°, indexed to (012), (104), and (110), respectively. The lattice constants can be calculated by the following equations:

(1) 2dsinθ=nλ,

(2) $$d_{hkl}={\left[{\left(\frac{h}{a}\right)}^{2}+{\left(\frac{k}{b}\right)}^{2}+{\left(\frac{l}{c}\right)}^{2}\right]}^{-\frac{1}{2}},$$

where *d* (*d_hkl_*) is the interplanar spacing, *θ* is the diffraction angle, *n* is the number of interplanar spacing, *λ* is the wavelength of X-rays (1.5406 Å), and *h*, *k*, and *l* are crystallographic indices. The calculated lattice constants are as follows: *a* = *b* = 5.5728 Å, *c* = 13.8852 Å, and agree with that of BFO NWs and bulk BFO in previous reports [28,29]. In addition, the crystal size can be calculated according to the Scherrer equation [30]:

(3) $$G=\frac{K\lambda }{\beta \cos\theta },$$

where *G* is the grain size (nm), *K* is the shape constant (*K* = 0.89), and *β* is the full width of the diffraction peak at half maximum. The calculation results show that the average crystal size is ~78.6 nm.

### 3.2. Ferroelectric Properties

In general, many static charges may accumulate at the surface of the sample as a consequence of the scanning process of piezoresponse force microscopy (PFM) mode, where a certain vibration may occur on both the piezoelectric effect and the electrostatic effect of the probe, which may disturb the PFM results. Also, previous studies have shown that the result of radial surface potential due to Fermi level pinning is caused by high density of surface states in BFO NWs [19]. Therefore, it is necessary to remove the electrostatic effect. Scanning Kelvin probe microscopy (SKPM) mode was adopted to measure the surface potential. After testing, the average surface potential is found to be about 1.2 V. In order to eliminate and compensate for the effect of static charges, an opposite tip voltage of 1.2 V was applied when characterizing the ferroelectric domains.

**Figure 2:**
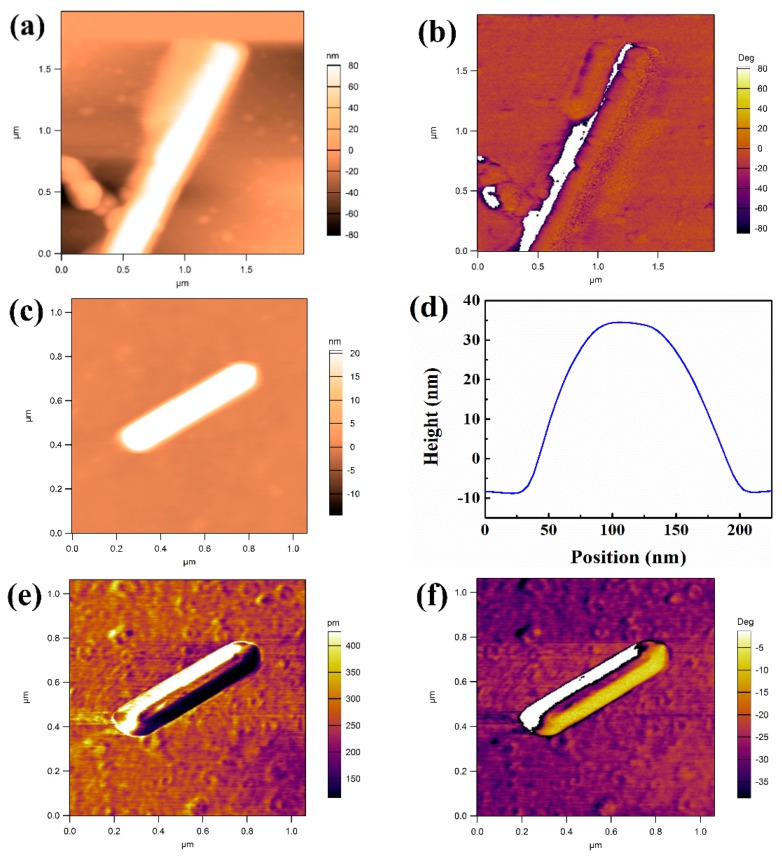
The topographies (**a**,**c**), phase (**b**,**f**), profile (**d**) and amplitude (**e**) images of two BFO nanowires (NWs).

BFO powder is composed of a large number of NWs concluded from atomic force microscope (AFM) mode (shown in Figure S1). The diameter and length of the NWs are inconsistent (the length and diameter range of BFO NWs are 200–4000 nm and 80–200 nm, respectively). The size is larger than the grain size, so the nanowires are polycrystalline. Figure 2 shows the topographies, phases, amplitude, and profile images that are tested by vertical PFM mode of two BFO NWs. From Figure 2a,c, it can be found that the diameter of the single NWs is uniform, and the diameters are 368.67 nm and 168.20 nm, respectively. The size of the NWs change after applying a voltage to the NWs. This may be due to the fact that the electrostatic attractive forces will cause a change in the topography (shown in Table S1). The PFM amplitude image and phase image of the NWs both exhibit ferroelectric domain structure. The amplitude of the background of the nanowires is taken as a reference. Part of the PFM amplitude is smaller than the amplitude of the background, which demonstrates that there is reverse deformation of the nanowires. In PFM, the phase of the response changes depending on the local domain orientation [31], that is, the phase change reflects the change of polarization direction. The PFM phase images (Figure 2b,f) show that the domain walls are throughout the as-grown NWs, which presents clear multi-polarization direction, indicating that there are at least two different types of the domain in single NW. According to the comparison of the polarization angles between domains, in this case, only 109° and 71° domains are possible in Figure 2c,d, respectively. 

**Figure 3:**
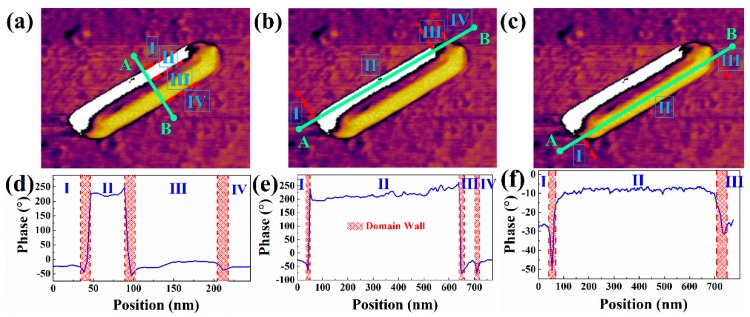
Thephase polarization direction versus position curves (**d**–**e**) and the corresponding position is marked in (**a**–**c**).

In order to intuitively show the change of polarization direction within and between domains, we took the NWs of Figure 2d as an example, and then three line segments were drawn in the figure to obtain the corresponding phase polarization direction versus position curves, as shown in Figure 3. It is obvious that there is a change of polarization direction at the junction of I, II and II, III in Figure 3d–f, and the width of the change area is from 15 to 40 nm. In comparison with Figure 3a–c, the change area corresponds to the domain wall. It is found that the domain wall is the transition region of polarization direction transition, and the width of the domain wall is different. The polarization direction of the domain walls is quite different from that of adjacent regions, and the polarization direction of the domain may be diverse in different regions (Figure 3f). In the phase images, the polarization direction within the domain is completely consistent and, in fact, there is a small angle deviation.

**Figure 4:**
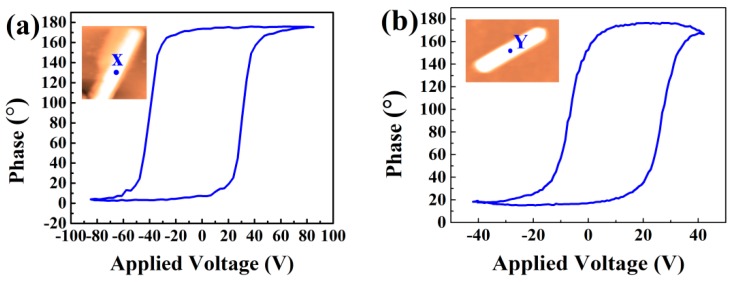
The local phase hysteresis loops of the BFO NWs ((**a**) corresponds to the NW in Figure 2a, while (**b**) corresponds to the NW in Figure 2b, and test points are marked in the inserted figure).

To further elucidate the ferroelectric domain switching mechanism of NWs, the local phase hysteresis loops were characterized by local switching spectroscopy piezoresponse force microscopy (SS-PFM) mode, as shown in Figure 4. These phase hysteresis loops reveal the change of the polarization direction with the voltage. As can be seen, the domain is almost stationary when the applied voltage is low. As the voltage increases gradually, the domain is only reversed over a small angle. When the voltage reaches a certain value, the domain appears to be greatly reversed. The domain switch angle is approximately 180°, while the applied voltages are 85 V and 42 V, respectively. As can be seen from the topographies in Figure 2a,b, the size of NW in Figure 2a is bigger than that in Figure 2b. Hence, it may mean that a higher voltage needs to be applied to achieve domain switch. Furthermore, the domain structure of the two NWs mentioned above may be different. This may also result in a different voltage being required.

### 3.3. Piezoelectric Property

**Figure 5:**
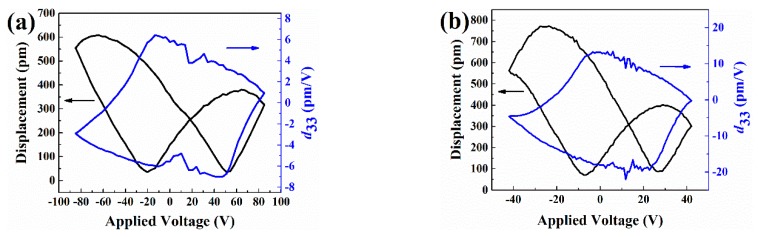
The butterfly and piezoelectric coefficients curves of BFO NWs ((**a**) and (**b**) are the results corresponding to NWs in Figure 2a,b, respectively).

Figure 5 shows the amplitude loops which demonstrate the electromechanical activity and the piezoelectric coefficient of the BFO NWs, wherein a typical butterfly shape was obtained. The butterfly curves are not symmetrical according to the *Y*-axis. The shift of the intersection may be due to the reason that the barrier between the NWs and PFM probe is different from that between the NWs and the bottom electrode. The deviation along the *X*-axis towards positive values is a characteristic of the imprint behavior of the local switching, and may be attributed to an internal built-in electric field which exists in the NWs [32]. Every point on the butterfly curve contains information about the piezoelectric deformation under the corresponding voltage. The relationship between displacement and applied voltage can be described as follows, according to the law of converse piezoelectric effect:

(4) $$D=d_{33}V,$$

where *D* is the displacement, *d*_33_ is the piezoelectric coefficient, and *V* is the applied voltage. However, since the intersection of the butterfly curve has an unexpected shift from the origin, the measured values of displacement and voltage should be modified. Thus, Equation (4) should be rectified to be [33]:

(5) $$D-D_{0}=d_{33}(V-V_{0}),$$

(6) $$d_{33}=\frac{D-D_{0}}{V-V_{0}},$$

where *D*_0_ and *V*_0_ are the displacement and voltage of the intersection. Through calculations, we obtained the piezoelectric coefficients versus voltage curves (blue lines in Figure 5). The maximum values of *d*_33_ are 6.53 pm/V and 22.21 pm/V in Figure 5a,b, respectively. The drive frequency is close to the resonant frequency of the probe sample in the test, and the values of *d*_33_ are affected. The transduction of any piezoelectric is enlarged near the resonance frequency [34]. Therefore, the actual values of *d*_33_ are smaller than the test results.

### 3.4. Electrical Conductivity

**Figure 6:**
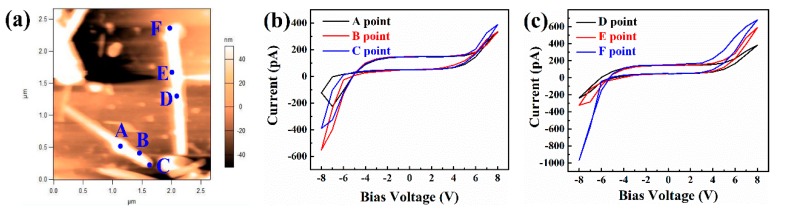
The topography (**a**) and *I*–*V* curves (**b**,**c**) of the BFO NWs at different positions.

Figure 6 shows the electrical properties of BFO NWs measured by conducting atomic force microscopy (C-AFM) mode. Six points (namely, the center, edge, and vertex of the NWs, as shown in Figure 6a) of the NWs were selected for testing, and the corresponding *I*–*V* curves are shown in Figure 6b,c. The current of the NWs is several hundred pA when the voltage is 8 V. However, the current of the NWs center is lower than that of the edge and vertex. The current at the vertex of the NW is the highest, and its values are 388.07 pA and 678.32 pA, respectively. We have three hypotheses for this result. First of all, we can know the edge and the vertex are thinner from the profile in Figure 2d, which possibly lead to the electrical properties change. Next, there are more defects in the edge and the vertex, resulting in larger current leakage. Then, it can be seen from Figure 2 that the edge and vertex of the NW are domain walls. Therefore, the edge and vertex of the NWs may also be domain walls in Figure 6. Hence, the current of the domain wall is higher than that of the domain. This may be due to the following reasons: Firstly, the charge at the domain wall brings about the accumulation of carriers, which can lead to a change in carrier concentration and then cause a change in conductivity. Secondly, it may be due to flexoelectric coupling and non-uniform strain [35]. Thirdly, the band gap of the domain wall is lower, which is mainly attributed to the shift of the conduction band [36]. The true explanation may also be a combination of these three guesses.

In addition, there is a significant hysteretic manner in the *I*–*V* curves. This proves that there is a contact barrier between NWs, and probe and electrode, and that the oxygen vacancy is usually present in BFO material. The oxygen vacancy is charged, so it will move in the electric field. Therefore, when the applied voltage direction changes, the oxygen vacancy will accumulate on different surfaces, thus affecting the upper and lower interface barriers and the transport at domain walls [37,38,39], and the polarization direction will be deflected under the field. The polarized head and tail are different, so even a slight change in direction can affect the barrier. Therefore, both of these factors will lead to the change of current at the same voltage, which will lead to the hysteresis of *I*–*V* curves.

## 4. Conclusions

In summary, the micro-zone performances of BFO NWs synthesized by hydrothermal method were investigated by SPM. The range of length and diameter of BFO NWs are 200~4000 nm and 80~200 nm, respectively. The NWs are of multidomain structure, and the domain can be switched about 180° when applying a certain voltage. The maximum *d*_33_ is 22.21 pm/V. The current is just hundreds of pA, and the current of the NWs center is lower than that of the edge and vertex. These results will improve our understanding of the micro-area performances of one-dimensional BFO NWs, laying the foundation for the application of BFO NWs.

## Supplementary Materials

The following are available online at http://www.mdpi.com/2079-4991/9/2/190/s1, Figure S1: The image of BFO NWs, Table S1: The deformation of the nanowires after applying voltage.

## References

[1] Åkerman J. (2005). Toward a universal memory. Science.

[2] Bai Z.L., Cheng X.X., Chen D.F., Zhang D.W., Chen L.Q., Scott J.F., Hwang C.S., Jiang A.Q. (2018). Hierarchical domain structure and extremely large wall current in epitaxial BiFeO_3_ thin films. Adv. Funct. Mater..

[3] Eerenstein W., Mathur N.D., Scott J.F. (2006). Multiferroic and magnetoelectric materials. Nature.

[4] Spaldin N.A., Cheong S.W., Ramesh R. (2010). Multiferroics: Past, present, and future. Phys. Today.

[5] Chappert C., Fert A., Van Dau F.N. (2007). The emergence of spin electronics in data storage. Nat. Mater..

[6] Zhai K., Shang D.S., Chai Y.S., Li G., Cai J.W., Shen B.G., Sun Y. (2018). Room-temperature nonvolatile memory based on a single-phase multiferroic hexaferrite. Adv. Funct. Mater..

[7] Sharma S., Tomar M., Kumar A., Puric N.K., Gupta V. (2016). Photovoltaic effect in BiFeO_3_/BaTiO_3_ multilayer structure fabricated by chemical solution deposition technique. Phys. Chem. Solids.

[8] Yang H., Jin C., Mi W.B., Bai H.L., Chen G.F. (2012). Electronic and magnetic structure of Fe_3_O_4_/BiFeO_3_ multiferroic superlattices: First principles calculations. J. Appl. Phys..

[9] Eliseev E.A., Morozovska A.N., Nelson C.T., Kalinin S.V. (2018). Intrinsic structural instabilities of domain walls driven by gradient couplings: Meandering anferrodistortive-ferroelectric domain walls in BiFeO_3_. arXiv.

[10] Chang M.L., Hu H.W., Zhang Y.Y., Chen D.C., Wu L.P., Li X.J. (2017). Improving visible light-absorptivity and photoelectric conversion efficiency of a TiO_2_ nanotube anode film by sensitization with Bi_2_O_3_ nanoparticles. Nanomaterials.

[11] Liu J., Zhou P., Han T., Huang J., Liu J., Li J., Braun P.V. (2018). Ni-encapsulated TiO_2_ nanotube array prepared using atomic layer deposition as a high-performance Li-ion battery anode. Mater. Lett..

[12] Cai Y.Y., Liu J.G., Tauzin L.J., Huang D., Sung E., Zhang H., Joplin A., Chang W.S., Nordlander P., Link S. (2018). Photoluminescence of gold nanorods: Purcell effect enhanced emission from hot carriers. Acs Nano.

[13] Serban E.A., Palisaitis J., Persson P.O.Å., Hultman L., Birch J., Hsiao C.L. (2018). Site-controlled growth of GaN nanorod arrays by magnetron sputter epitaxy. Thin Solid Film..

[14] Jevasuwan W., Chen J., Subramani T., Pradel K.C., Takei T., Dai K., Shinotsuka K., Hatta Y., Fukata N. (2017). Pencil-shaped silicon nanowire synthesis and photovoltaic application. Jpn. J. Appl. Phys..

[15] Huang B.R., Hung S.C., Hsu C.H., Tu C.W., Yang W.L. (2016). Ultra-low reflection loss for silicon nanowire-array-textured based photovoltaic devices. Mater. Res. Bull..

[16] Lei D., Benson J., Magasinski A., Berdichevsky G., Yushin G. (2017). Transformation of bulk alloys to oxide nanowires. Science.

[17] Ali S.S., Li W.J., Javed K., Shi D.W., Riaz S., Zhai G.J., Han X.F. (2016). Exchange bias in two-step artificially grown one-dimensional hybrid Co-BiFeO_3_ core-shell nanostructures. Nanotechnology.

[18] Lei Y., Zeng H.Z., Luo W.B., Shuai Y., Wei X.H., Du N., Bürger D., Skorupa I., Liu J.S., Schmidt O.G. (2016). Ferroelectric and flexible barrier resistive switching of epitaxial BiFeO_3_ films studied by temperature-dependent current and capacitance spectroscopy. J. Mater. Sci. Mater. Electron..

[19] Prashanthi K., Dhandharia P., Miriyala N., Gaikwad R., Barlage D., Thundat T. (2015). Enhanced photo-collection in single BiFeO_3_ nanowire due to carrier separation from radial surface field. Nano Energy.

[20] Chybczyńska K., Markiewicz E., Błaszyk M., Hilczer B., Andrzejewski B. (2016). Dielectric response and electric conductivity of ceramics obtained from BiFeO_3_ synthesized by microwave hydrothermal method. J. Alloys Compd..

[21] Yu P., Wu J., Liu S., Xiong J., Jagadish C., Wang Z.M. (2016). Design and fabrication of silicon nanowires towards efficient solar cells. Nano Today.

[22] Prashanthi K., Antić Ž., Thakur G., Dramićanin M.D., Thundat T. (2018). Surface state-induced anomalous negative thermal quenching of multiferroic BiFeO_3_ nanowires. Phys. Status Solidi–R.

[23] Liu J., Prashanthi K., Li Z., McGee R.T., Ahadia K., Thundat T. (2016). Strain-induced electrostatic enhancements of BiFeO_3_ nanowire loops. Phys. Chem. Chem. Phys..

[24] Biswas K., De D., Bandyopadhyay J., Dutta N., Rana S., Sen P., Bandyopadhyay S.K., Chakrabortye P.K. (2017). Enhanced polarization, magnetic response and pronounced antibacterial activity of bismuth ferrite nanorods. Mater. Chem. Phys..

[25] Prashanthi K., Shaibani P.M., Sohrabi A., Natarajan T.S., Thundat T. (2012). Nanoscale magnetoelectric coupling in multiferroic BiFeO_3_ nanowires. Phys. Status Solidi–R.

[26] Prashanthi K., Gaikwad R., Thundat T. (2013). Surface dominant photoresponse of multiferroic BiFeO_3_ nanowires under sub-bandgap illumination. Nanotechnology.

[27] Li S., Nechache R., Harnagea C., Nikolova L., Rosei F. (2012). Single-crystalline BiFeO_3_ nanowires and their ferroelectric behavior. Appl. Phys. Lett..

[28] Patel S.K.S., Lee J.H., Kim M.K., Bhoi B., Kim S.K. (2018). Single-crystalline Gd-doped BiFeO_3_ nanowires: R3c-to-Pn2_1_a phase transition and enhancement in high-coercivity ferromagnetism. J. Mater. Chem. C.

[29] Marzouki A., Harzali H., Loyau V., Gemeiner P., Zehani K., Dkhil B., Bessais L., Megriche A. (2018). Large magnetoelectric response and its origin in bulk Co-doped BiFeO_3_ synthesized by a stirred hydrothermal process. Acta. Mater..

[30] Muniz F.T.L., Miranda M.A.R., dos Santos C.M., Sasaki J.M. (2016). The Scherrer equation and the dynamical theory of X-ray diffraction. Acta Cryst. A.

[31] Vasudevan R.K., Jesse S., Kim Y., Kumar A., Kalinin S.V. (2012). Spectroscopic imaging in piezoresponse force microscopy: New opportunities for studying polarization dynamics in ferroelectrics and multiferroics. MRS Commun..

[32] Gruverman A., Kholkin A., Kingon A., Tokumoto H. (2001). Asymmetric nanoscale switching in ferroelectric thin films by scanning force microscopy. Appl. Phys. Lett..

[33] Dong H., Zheng X.J., Li W., Gong Y.Q., Peng J.F., Zhu Z. (2011). The dielectric relaxation behavior of (Na_0.82_K_0.18_)_0.5_Bi_0.5_TiO_3_ ferroelectric thin film. J. Appl. Phys..

[34] Ren B., Or S.W., Zhao X., Luo H. (2010). Energy harvesting using a modified rectangular cymbal transducer based on 0.71Pb(Mg_1/3_Nb_2/3_)O_3_–0.29PbTiO_3_ single crystal. J. Appl. Phys.

[35] Seidel J., Vasudevan R.K., Valanoor N. (2016). Topological structures in multiferroics–domain walls, skyrmions and vortices. Adv. Electron. Mater..

[36] Chiu Y.P., Chen Y.T., Huang B.C., Shih M.C., Yang J.C., He Q., Liang C.W., Seidel J., Chen Y.C., Ramesh R. (2011). Atomic-scale evolution of local electronic structure across multiferroic domain walls. Adv. Mater..

[37] Seidel J., Martin L.W., He Q., Zhan Q., Chu Y.H., Rother A., Hawkridge M.E., Maksymovych P., Yu P., Gajek M. (2009). Conduction at domain walls in oxide multiferroics. Nat. Mater..

[38] Seidel J., Maksymovych P., Batra Y., Katan A., Yang S.Y., He Q., Baddorf A.P., Kalinin S.V., Yang C.H., Yang J.C. (2010). Domain Wall Conductivity in La-Doped BiFeO_3_. Phys. Rev. Lett..

[39] Fan W., Cao J., Seidel J., Gu Y., Yim J.W., Barrett C., Yu K.M., Ji J., Ramesh R., Chen L.Q. (2011). Large kinetic asymmetry in the metal-insulator transition nucleated at localized and extended defects. Phys. Rev. B.

