# Supplementary material for: Micro-Area Ferroelectric, Piezoelectric and Conductive Properties of Single BiFeO3 Nanowire by Scanning Probe Microscopy
Source: Nanomaterials (Basel). 2019 Feb 2;9(2):190. doi: 10.3390/nano9020190 (PMC6409863; doi:10.3390/nano9020190)
Supplement: Supplementary file 1 [file nanomaterials-09-00190-s001.pdf]

Micro-area ferroelectric, piezoelectric and conductive properties of single $BiFeO_3$ nanowire by scanning probe microscopy

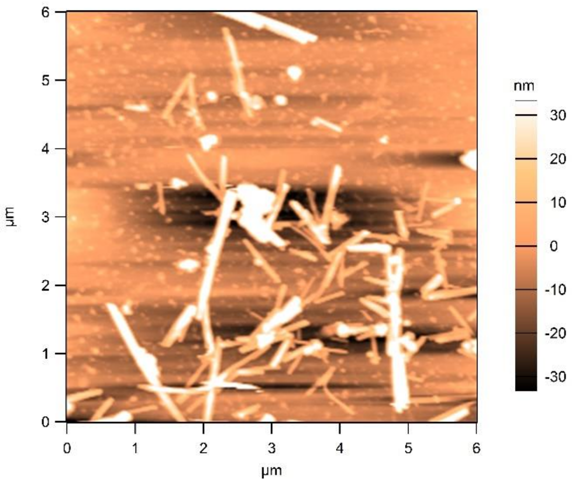

Figure S1. The image of BFO NWs.

Table S1. The deformation of the nanowires under voltage.

Sample	Diameter (nm) (before applying voltage)	Diameter (nm) (after applying voltage)	Deformation rate
NW in Figure 2a	368.67	381.57	3.51%
NW in Figure 2b	168.20	173.53	3.16%
